# Parasitémie asymptomatique chez les enfants de moins de 5 ans, enfants en âge scolaire et prise en charge des épisodes fébriles dans les ménages de Lubumbashi, République Démocratique du Congo

**DOI:** 10.11604/pamj.2016.24.94.9350

**Published:** 2016-05-27

**Authors:** Sompwe Eric Mukomena, Cilundika Mulenga Philipe, Mashinda Kulimba Désiré, Lutumba Tshindele Pascal, Mapatano Mala Ali, Luboya Numbi Oscar

**Affiliations:** 1 Département de Santé Publique, Faculté de Médecine, Université de Lubumbashi, République Démocratique du Congo; 2 Ecole de Santé Publique, Université de Lubumbashi, République Démocratique du Congo; 3 Ecole de Santé Publique, Université de Kinshasa, République Démocratique du Congo; 4 Département de Médecine Tropicale, Faculté de Médecine, Université de Kinshasa, République Démocratique du Congo

**Keywords:** Paludisme asymptomatique, enfants de moins de 5 ans et en âge scolaire, automédication, Asymptomatic paludism, children of less than 5 years and school age children, self-medication

## Abstract

**Introduction:**

longtemps négligé, le paludisme asymptomatique est actuellement reconnu comme potentielle menace et frein au contrôle du paludisme. En RD Congo, la prévalence de cette parasitémie est peu documentée. L'objectif de cette étude était de déterminer la prévalence de la parasitémie asymptomatique aussi bien chez les enfants de moins de 5 ans que ceux âgés de plus de cinq ans aux regards des interventions de masse en cours (MILDS).

**Méthodes:**

il s'agit d'une étude transversale menée chez les écoliers et chez les enfants de moins de cinq ans dans les ménages de Lubumbashi. Les écoles, les écoliers et les enfants de moins de 5 ans avaient été sélectionnés aléatoirement. Les frottis, gouttes épaisses et les tests rapides avaient été prélevés et lues.

**Résultats:**

sur 350 écoliers examinés, 43 soit une prévalence de 12,3%, IC 95% (9,14-16,04) avaient une goutte épaisse positive. Dans tous les 43 cas, seul le Plasmodium falciparum a été identifié.314 ménages, soit 90,5% ont déclaré avoir recouru aux antipaludéens pour soigner la fièvre de leurs enfants à domicile. Plus d'1/3 des enfants dans les ménages, soit 39,9% des interviewés avait avoué avoir recouru aux antipyrétiques pour soulager la fièvre de leurs enfants, 19,7% à la quinine et seulement moins de 2% aux ACT à base de Lumefantrine. En considérant l'utilisation des TDR, la prévalence due à la parasitémie asymptomatique est de 3% IC 95% (2,075-4,44), mais quand on considère la microscopie comme le gold standard, cette prévalence est de 1,9%, IC 95% (1,13-3,01).

**Conclusion:**

le paludisme asymptomatique n'est pas exempt de toute conséquence sur la santé, il est donc important de mener des enquêtes pareilles pour identifier les nouvelles stratégies de contrôle du paludisme.

## Abstract (en)

**Introduction:**

Long neglected, asymptomatic malaria is currently recognized as a potential threat and obstacle to malaria control. In DR Congo, the prevalence of this parasite is poorly documented. This study aims to determine the prevalence of asymptomatic parasitaemia in children less than 5 years of age as well as in those aged over five years for what concerns ongoing mass control interventions (LLINs).

**Methods:**

This is a cross-sectional study conducted among school age children, children less than 5 years of age living in the household of Lubumbashi. Schools, students and children less than 5 years of age were selected randomly. Thick and thin blood smears and rapid tests were performed and read.

**Results:**

Out of 350 examined students, 43 (12, 3%), IC 95% (9, 14-16, 04) had positive thick smear. Only plasmodium falciparum was identified in all the 43 cases. 314 households (90.5%) declared that they had administered anti-malarial drugs to their children to treat fever at home. More than one-third of households (39.9%) declared that they had administered antipyretics to their children to relieve fever, 19.7% administered quinine and only less than 2% artemether-lumefantrine. Considering the use of the TDR technique, the prevalence of asymptomatic parasitaemia was 3%, IC 95% (from 2.075 to 4.44), but if we consider microscopy as the gold standard, the prevalence was 1.9%, IC 95% (from 1.13 to 3.01).

**Conclusion:**

Asymptomatic malaria is not without health consequences, so it is important to conduct such investigations to detect new malaria device programmes.

## Introduction

L'Organisation Mondiale de la Santé (OMS) estime qu'il y a eu dans le monde 198 millions de cas de paludisme (127 millions-283 millions) et 584 milles décès. Plus de 90% de cas et au moins Quatre-vingt pourcents de ces décès sont survenus en Afrique en dessous du Sahara [1]. Comme stratégies utilisées pour le contrôle de cette endémie tropicale, nous pouvons citer entre autres: la distribution de masse de la moustiquaire imprégnée d'insecticide à longue durée (MILD), la communication inter personnelle pour améliorer la couverture de son utilisation, la lutte anti vectorielle, le diagnostic précoce et la prise en charge précoce des cas avec des médicaments efficaces comme les combinaisons à base d'artémicine [2–4]. Les enfants de moins de cinq ans et les femmes enceintes demeurent des groupes vulnérables dans les régions à transmission modérée ou intense [2, 5–7]. Toutefois dans les zones d'endémie palustre instable, les auteurs rapportent que les enfants d’âge scolaire ainsi que les adultes constituent d'importants groupes à risque aussi bien de paludisme asymptomatique que de paludisme clinique [8–10]. A l'heure du passage à l’échelle des interventions à haut impact sur l'ampleur du paludisme, la persistance de la parasitémie asymptomatique dans la population autochtone est inquiétante car elle pourrait constituer un réservoir, une menace à l'atteinte de l’élimination du paludisme [11, 12]. Nonobstant, les efforts de contrôle, la nature de la transmission, la géographie de la zone ainsi que le type d'habitat pourraient déterminer l'ampleur du paludisme [8, 13, 14]. En dépit de la faible qualité des données décriée régulièrement par l'Organisation Mondiale de la Santé à l’égard des certains pays Africains, comme la RDC [1–3], les cas rapportés ne cessent d'augmenter; ceci pourrait se comprendre aisément car l'accès aux soins s'est considérablement améliorée, comme en témoigne la spectaculaire diminution de la mortalité infantile. Malheureusement, en RDC et particulièrement à Lubumbashi, les informations sur la prévalence du paludisme asymptomatique des enfants de moins de cinq ans et ceux en âge scolaire sont très peu disponibles. Quand elles existent, elles ont de l’âge et donc peu exploitables [15, 16].

## Méthodes

L’étude s'est déroulée dans la ville de Lubumbashi, au Sud Est de la République Démocratique du Congo. Du point de vue sanitaire, la ville compte 9 districts sanitaires, 7 Hôpitaux généraux de référence et 104 centres de santé appartenant majoritairement aux privés [17]. Cette ville où vivent près de deux millions d'habitants, a une économie basée essentiellement sur l'extraction minière et l’économie informelle. Le paludisme y est endémique [18]. Deux enquêtes transversales ont été conduites: la première a ciblé les enfants en âge scolaire dans neuf écoles primaires de la ville. Quant à l'enquête ménage, elle a ciblé les enfants de moins de 5 ans des Huit districts sanitaires. Ces enquêtes se sont déroulées en 2013, en pleine saison sèche, du mois de Mai à la fin du mois de Juin.

**Echantillonnage:** Se basant sur une étude antérieure dans le milieu scolaire, la prévalence du paludisme asymptomatique de 34%. Ainsi, la taille retenue pour cette première partie de l’étude était de 380 enfants. Nous avions procédé à un échantillonnage à plusieurs degrés, successivement: le l’école, ensuite la classe et enfin de l’élève. L’école a été sélectionnée sur base de la liste préétablie au ministère de l'enseignement primaire et secondaire. Au sujet de la taille de l'enquête de la parasitémie asymptomatique dans les ménages, il était de convenance, et devait être plus que le double de celui des écoles. C'est ainsi que nous avons travaillé sur un échantillon de 880 enfants de moins de cinq ans. Ici aussi l’échantillonnage aléatoire était appliqué(aire de santé, avenue, maison, ménage et l'enfant. Les enquêteurs étaient tous de niveau universitaire et avaient tous été formés. Pour la partie laboratoire, 3 laborantins expérimentés du laboratoire provincial de référence ont contribué à la collecte et à l'analyse des échantillons. Dans la partie qui porte sur les ménages, nous avions utilisé seulement la goutte épaisse et le frottis mince. Les échantillons étaient préparés sur la même lame en utilisant un code pour chaque patient. Ils ont été colorés avec une solution de Giemsa. La lecture était faite au laboratoireProvincial de Référence. Deux différents lecteurs à simple aveugle ont lu et en cas de discordance un troisième lecteur, lui à double aveugle faisait la lecture. Le Test rapide du diagnostic du paludisme qui a été utilisé pour les moins de cinq ans, était à base d'antigène Plasmodium falciparum: Histidine Rich Protein-II et pLDH (Lactate Deshsydrogenase.f/Pan), SD Bioline, dont la sensibilité est de 93.8- 99,7%. Les enquêteurs formés et supervisés ont réalisé ce test. L'examen microscopique était fait par des personnes expertes et contrôlées par une équipe accréditée par le NICD (National Institute Control Diseases). Il était conforme aux procédures de l'OMS pour l'examen microscopique du paludisme et adoptées en RD Congo [19]. Deux questionnaires pré testés ont servi à collecter les informations aussi bien dans les ménages que dans les écoles. L'enquête a été précédée de l'obtention du consentement éclairé des parents ou responsables légaux des enfants.

**Analyses des données:** Les données collectées ont été encodées, traitées et analysées à l'aide du logiciel SPSS version 21(Chicago software package). Le logiciel Epi open a aussi servi pour certaines analyses dont la détermination des odd ratio, le test de Khi carré. Pour montrer la différence entre l'utilisation de la MII et/ou la non utilisation de la moustiquaire imprégnée d'insecticide comme facteur prédictif de risque de paludisme, nous avons utilisé le test de Khi2 et l'intervalle de confiance.

## Résultats

**Les données socio démographiques de l'enquête dans les écoles et dans les ménages:** La taille de l’échantillon dans les écoles était de 364 sujets (écoliers âgés de 6 à 15 ans) mais 13 écoliers ont refusé la prise de sang pour la parasitémie. Nous avons par la suite travaillé avec 350 écoliers, dont 121 écoliers de sexe féminin, soit 34,6% et 229 écoliers de sexe masculin, soit 65,4%. L’âge moyen des écoliers était de 9,3 ans, l’âge minimal était de 6,9 ans. L’âge maximum, quant à lui était de 15 ans. La taille de l’échantillon dans les ménages était de 840 enfants; estimé à 900, en partant de l'hypothèse qu'elle devait représenter plus que le double de la prévalence du paludisme chez les écoliers, 8 72 enfants ont pu participer à l'enquête. Des ceux-ci, 872 échantillons ont été prélevés, 32 n’étaient pas interprétables du fait soit de l'illisibilité du nom ou de mauvais étalement et donc, ils ont dû être déclassés pour rester avec 840 enfants. Trois cent nonante deux enfants étaient de sexe masculin, soit 46,7% et 448 enfants de sexe féminin, soit 53,3%. L’âge moyen pour les filles était de 2,5 années et pour les enfants de sexe masculin, il était de 2,6 années. Chez tous les 840, la seule espèce retrouvée était le plasmodium falciparum.

**Densité parasitaire chez les écoliers et dans les ménages:** 5 écoliers sur 46, soit 11,6% avaient une parasitémie supérieure à 2000, tandis que 40% ont une parasitémie inférieure à 2000 parasite par microlitre de sang. La densité parasitaire moyenne était de 1527. Tandis que les densités parasitaires minimale et maximale étaient respectivement de 244 et de 7269 (Tableau 1). Il y a association positive entre le fait de dormir sous MILD et une parasitémie, OR= 0,44 mais cette association n'est pas statistiquement significative au seuil de 95% (Tableau 2). En considérant l'utilisation des TDR, la prévalence due à la parasitémie asymptomatique est de 3% IC 95% (2,075-4,44), mais quand on considère la microscopie comme le gold standard, cette prévalence est de 1,9%, IC 95% (1,13-3,01) (Tableau 3). Aucun de deux groupes d’âge n'est protégé de façon statistiquement significative contre la parasitémie palustre asymptomatique, p> 0,05 (Tableau 4). Les enfants ayant passé nuit sous MILD sont 5 fois plus protégés contre le paludisme que Ceux qui ne passent pas nuit sous MILD au seuil de signification de 95% (Tableau 5).

**Tableau 1:** Distribution de la densité parasitaire palustre chez les écoliers de Lubumbashi

Densité parasitaire	Effectif	Pourcentage (%)
<500	10	23,2
500-1000	12	27,9
1000-2000	16	37,3
>2000	5	11,6
**Total**	**43**	**100**

**Tableau 2:** Utilisation de la moustiquaire imprégnée et risque par catégorie d’âge

Age	6-9 ans		Total	OR	Pvalue	10- 15 ans		Total	OR	Pvalue
	Goutte épaisse positive	Goutte épaisse négative	-	-	-	Goutte épaisse positive	Goutte épaisse négative	-	-	-
A	9	58	67	0.44 (0,13-1,4)	0,16	17	63	80	4,15 (1,72-10,8)	0,0028
N'a	6	17	23	-	-	11	169	180	-	-
Total	15	75	90	-	-	28	232	260	-	-

**Tableau 3:** Prévalence du Paludisme asymptomatique chez les enfants de moins de 5 ans dans la ville de Lubumbashi

	Microscopie			Kappa	IC (95%)
TDR	Négatif	798	0	798	0,54 (0,079-17,6)
	Positif	26	16	42	-
Total		824	16	840	-

**Tableau 4:** Catégories d’âge, microscopie positive et passer nuit sous moustiquaire imprégnées d'insecticide à longue durée

Age groupe	Microscopie			OR IC (95%)	Passe nuit Sous MILD			OR IC (95%)
	Négatif	Positif	Total	2,7(0,36-20,9)	Oui	Non	Total	1,95 (0,45-8,6)
≤ 1 an	127	1	128	-	95	2	98	-
≥ 5 ans	697	15	712	-	466	19	485	-
Total	824	16	840	-	562	21	583	-

**Tableau 5:** Risque de portage palustre asymptomatique et utilisation de la moustiquaire imprégnée d'insecticide à longue durée

	Parasitémie positive	Parasitémie négative	Total	OR IC 95%	P-Valu
A dormi S/MILD	12	197	209	0,2 (0,076-0,5)	0,0027
pas dormi s/MILD	9	29	38	-	-
Total	21	226	247	-	-

**Prise en charge de la fièvre à domicile:** Sur 840 enfants, 213 avaient connu l'automédication dans les deux semaines qui avaient précédées l'enquête. De ces 213, plus du tiers, soit 39,9% des interviewés ont avoué avoir recouru aux antipyrétiques pour soulager la fièvre de leurs enfants, 19,7% à la quinine et seulement moins de 2% aux ACT à base de Lumefantrine. Il est aussi intéressant des noter que près de 10 enfants sur 213, soit 5% avaient bénéficiés d'une antibiothérapie comme traitement de la fièvre à domicile. En ce qui concerne, l'administration des antipaludéens, sur 213 enfants automédiqués, 47 ont reçu les antis paludéens, soit 22%. Parmi les antipaludéens administrés aux enfants par leurs parents, 42/47, soit 89,3% étaient la quinine. Les ACT ne représentent que moins de 20% (Tableau 6).

**Tableau 6:** Molécules utilisées pour l'automédication dans les ménages de Lubumbashi

Médicament	Effectif	Pourcentage	Observation
Antipyrétique(ATP)	85	39,9	
Anti inflammatoire non stéroïdien (AINS)	24	11,26	
Antibiotique(ATB)	4	1,9	
AINS+ATB	3	1,4	
ATP + ATB	3	1,4	
AMODIAQUINE	1	0,46	
ACT	3	1,4	A base de Lumefantrine
ACT +APP	1	0,46	A base de Lumefantrine
QUININE	42	19,7	
Ne se rappelle plus	47	22	
Total	213	10	

## Discussion

En effet, sur 350 écoliers enquêtés, cette étude a trouvé que 46, soit 16% d'entre eux avaient une parasitémie positive au paludisme. Ces résultats sont comparables à ceux rapportés dans un des trois districts de la Tanzanie. En, effet, une étude rapporte, une prévalence du paludisme asymptomatique de 20% à Thyolo. Mais sont nettement supérieurs à ceux des deux autres districts qui sont respectivement de 5, et 10% à Blantyre et Chikhwawa, au Malawi [10]. En Gambie, en Ethiopie et au Kenya, les enquêtes menées ont montré des prévalences plus basses que les nôtres. Dans certaines régions de ces deux pays, surtout les régions frontalières suite au mouvement des populations des pays à forte transmission vers la Gambie, sans dépasser nos résultats, la parasitémie était supérieure à 10% [20–22]. Notre étude rejoint celle du Kenya car cette dernière a révélé que la tranche d’âge de 5 ans à 15 ans est plus touchée que celle des enfants de moins de cinq ans. En revanche, une autre étude menée dans ce même pays, a trouvé un portage asymptomatique de 13% [23]. Mais le Kenya a un paysage épidémiologique différent de la RDC. En Tanzanie et au Bhutan, en Asie, c'est une prévalence nulle que les études ont mise en évidence. Ces résultats sont attribués dans les deux études à la couverture et au taux d'utilisation des MILD, très élevés, dans un contexte de pré-élimination du paludisme [24, 25]. Ensuite, la période à laquelle on conduit l’étude pourrait aussi expliquer les différences observées. Les auteurs lient cette situation comme ceux cités précédemment au paludisme trans-frontalier et à l'acquisition de l'immunité [26]. Contrairement aux enfants de plus de cinq ans, La prévalence du paludisme asymptomatique chez les enfants de moins de 5 ans est surprenante. Seulement 1,9% des 840 enfants ont une microscopie positive. Ce résultat ne saurait s'expliquer que par des couvertures élevées en MILD et leur utilisation effective [27, 28]. En effet, cette enquête intervient seulement 8 mois après la campagne de masse de distribution des MILD. La forte sensibilisation des ménages pourrait expliquer la forte adhésion, surtout que les MILD ont été distribuées pendant la saison d'intense transmission du paludisme. Au Rwanda, deux différentes études: l'une, dans une zone avec des couvertures en MILD et IPD, toutes 2 supérieures à 90%, les porteurs asymptomatique ont représenté. Dans l'autre étude qui s'est déroulée dans une zone à endémie stable, les auteurs rapportent, une microscopie palustre asymptomatique dans la communauté de près de 11% [29]. A Kinshasa, en RDC, une autre étude récente a trouvé des résultats 7 fois plus élevés que les nôtres [30]. La différence de prévalence observée entre les deux techniques de diagnostic, d'une part la microscopie et d'autre part le TDR, s'expliquerait par l'effet prozone, décrite partout ailleurs ou les réactions croisées avec d'autres anticorps [31, 32]. Autant pour les enfants de moins de cinq ans que pour ceux âge scolaire, utiliser et passer nuit sous la MILD avait un effet protecteur, néanmoins, cette protection s'est montrée statistiquement non significatif (p > 0,05). Il est tout de même bien de noter que le taux de possession de la MILD et de son utilisation par les enfants de ces deux groupes d’âge étudiées est en nette progression. La proportion des ménages possédant la MILD dans les deux groupes est la même, 70% pour les enfants d’âge scolaire et 69,4% pour les moins de cinq ans. Par contre la proportion des enfants qui passent nuit sous MILD est significativement élevée, elle est de 96,4% comparée à 61,7%. L'enquête organisée après distribution de la MILD rapporte respectivement des taux d'utilisation de MILD post campagne de distribution de cette dernière. Ceci, appelle à plus d'attention chez les enfants d’âge scolaire. Il est bien connu comme rapporté partout ailleurs que ce taux est élevé chez les enfants de moins de cinq ans et que ceux-ci seraient très vulnérables comparé aux enfants d’âge scolaire [33, 34].

Densité parasitaire chez les écoliers et les enfants de moins de 5 ans dans les ménages. La densité parasitaire chez les 43 écoliers est comprise entre 200 et 7600 parasites/ul de sang. Parmi ces derniers, 38 soit, 88,4% avaient une parasitémie comprise entre 200 et 2000. Seuls 5 écoliers avaient une parasitémie supérieure aux normes de l'Organisation Mondiale de la Santé, en zone de haute transmission. Ces chiffres sont largement bas que ceux reconnus et universellement acceptés en zone d'endémie palustre où il est commun de voir des personnes asymptomatiques avec des parasitémies élevées. En côte d'Ivoire dans la ville d'Abidjan, une étude rapporte des parasitémies dépassant 25000 chez au moins 32% de la population d’étude [35]. De même, Rougemont, au Niger, rapporte des parasitémies allant jusqu’à 100.000 chez des enfants totalement apyrétiques [36]. Dans ce travail, nous avons trouvé qu'au moins 12% d’écoliers avaient une parasitémie supérieure à 2000. L'on pourrait dire que cette proportion est faible pour entretenir la transmission si elle était extrapolable à la population générale, mais ceci n'est pas le cas. L'utilisation à large échelle des MILD et l'utilisation des ACT pourraient expliquer nos résultats [37–39]. Prenant en compte les conséquences de la parasitémie asymptomatique sur le développement cognitif de l'enfant dans le futur, cette prévalence parait élevée [40].

**Prise en charge de la fièvre à domicile**. Avec une parasitémie inférieure à 2% chez les moins de cinq ans, ce travail révèle que lors des épisodes fébriles antérieurs attribués au paludisme, les responsables de ces enfants leur ont administré une auto médication dans 22% des cas. La quinine était utilisée dans 42/47 cas, soit, 89,3% IC (77,9-96). Sur le continent Africain, une étude rapporte des proportions plus élevés d'automédication (74%). Majoritairement, les ménages avaient recouru aux analgésiques et à la chloroquine [41]. Cependant, nos résultats sont de même de loin meilleur que ceux rapportés au Nigéri [42]. Il apparait aussi que plus jeune est l'enfant, moins les parents appliquent l'automédication anti palustre, peut être connaissent ils le risque qu'encoure l'enfant? Les parents ou responsables des enfants amèneraient plus probablement ces enfants dans un système de soins formels,à en croire, la faible proportion d'automédication. L'automédication des enfants âgés de plus de 5 ans, quant à elle, se fait massivement avec les antipaludéens (>90%). La quinine a été le médicament de choix et les combinaisons thérapeutiques à base d'artemycinine ont été le choix dans 10,8%. Cette ampleur de l'automédication chez les écoliers est similaire aux résultats d'une étude Tanzanienne. Presque tous les participants à cette étude, avaient avoué pratiquer l'automédication. La même étude révèle que la population a continué à utiliser la SP malgré le changement de la politique de prise en charge du paludisme [43]. Ceci corrobore avec notre étude dans laquelle, la SP, 8 ans après révision de la politique nationale, cette molécule continue d’être utilisée aux cotés de la monothérapie, quoique dans des proportions négligeables, et cela plusieurs années après changement de la politique nationale [44]. Le coût relativement bas et la simplicité de la posologie de la SP pourraient expliquer le recourt à ce dernier.

## Conclusion

L'objectif principal de cette étude était de mesurer l'ampleur de la parasitémie asymptomatique chez les enfants de moins de 5 ans et ceux en âge scolaire. La majorité absolue des infections est causée par le plasmodium falciparum. La densité parasitaire chez les écoliers est basse, elle est en générale inférieure au seuil parasitemique considéré par l'OMS comme pathologique en Zone holo-endémique. La distribution universelle de la MLID pourrait jouer un grand rôle dans la faible prévalence observée mais, cette étude n'a démontré aucune association significative entre la parasitémie asymptomatique et l'utilisation des MILD. Le paludisme asymptomatique est plus prononcé chez les écoliers que chez les moins de 5 ans. Quelques mois après la distribution universelle de la MILD dans la ville, les taux de possession ainsi que celui d'utilisation demeure encore moyens quoique amélioré par rapport aux années antérieures. L'automédication en présence d’épisode fébrile affecte encore bon nombre des ménages et la quinine continue à être utilisée abusivement. Cette dernière est relativement bonne chez les enfants de moins de cinq ans.

### Etat des connaissances actuelle sur le sujet

- En zones endémiques, les enfants de moins de cinq ans sont un groupe le plus vulnérable;
- Les enfants de moins de cinq ans constituent la cible majeure des interventions stratégiques du contrôle du paludisme;
- La protection des enfants de moins de cinq ans contre le paludisme est souvent la raison expliquant l'utilisation des moustiquaires.

### Contribution de notre étude à la connaissance

- Plus d'attention doit être accordée aux enfants en âge scolaire dans la prévention;
- On doit accorder aussi l'attention aux enfants en âge scolaire dans la prise en charge du paludisme dans les ménages;
- Le plasmodium *falciparum* est l'unique espèce retrouvée Lubumbashi.
